# Dietary nutrients and health risks from exposure to some heavy metals through the consumption of the farmed common carp (*CYPRINUS CARPIO*)

**DOI:** 10.1007/s40201-021-00647-4

**Published:** 2021-03-19

**Authors:** Halyna Tkachenko, Natalia Kurhaluk, Olha Kasiyan, Piotr Kamiński

**Affiliations:** 1 Institute of Biology and Earth Sciences, Pomeranian University in Słupsk, Arciszewski Str. 22b, 76-200 Słupsk, Poland; 2 Danylo Halytskyy Lviv National Medical University, Lviv, Ukraine; 3 Collegium Medicum in Bydgoszcz, Department of Medical Biology and Biochemistry, Department of Ecology and Environmental Protection, Nicolaus Copernicus University in Toruń, M. Skłodowska-Curie St. 9, Bydgoszcz, PL 85-094 Poland; 4 Faculty of Biological Sciences, Department of Biotechnology, University of Zielona Góra, Prof. Z. Szafran St. 1, Zielona Góra, PL 65-516 Poland

**Keywords:** Fish, Dietary nutrients, Toxicity, Target hazard quotients, Health risk, Carcinogenic risk

## Abstract

Common carp (*Cyprinus carpio*) is one of the most important cultured fish species in Poland. The aims of our study were to determine the concentration of essential minerals in the muscle tissue of carp obtained from a pond culture and to compare the content of these minerals with the physiological norms of nutrition for the Polish population, Recommended Dietary Allowances (RDA), and Estimated Average Requirements (EAR). The non-carcinogenic and carcinogenic risk by assessment of the Estimated target hazard quotients (THQ), total target hazard quotients (ΣTHQ), and carcinogenic risk were also studied. The muscle tissue of the carp was rich in macroelements. The pattern significance gradation of element concentrations was as follows: Na > K > Ca > P > Mg > Al > Zn > Fe > Cu > Mn > Cr > Se > Co. The concentration of toxic heavy metals in the samples was lower than the maximum allowable level of metals in food products. The risk of consumption of toxicants by an adult at a daily intake of 100 g of fish was 1.45% for Pb, 0.1% for Hg, 0.02% for Cd, and 0.02% for As, compared to the allowable daily intake for each toxicant. The value of the target hazard quotients of each metal was less than 1, indicating that the intake of a single metal through the consumption of carp meat does not pose a considerable health hazard. The total value of the target hazard quotients was also less than 1, indicating the safety of the combined effects of the chemicals. The major risk contributor was Pb with 82.9%, followed by Hg (9.38%), As (6.43%), and Cd (1.29%).

## Introduction

Fish, as human food, is considered a good source of proteins, polyunsaturated fatty acids (particularly omega-3 fatty acids), essential vitamins, and minerals [6, 11]. In the future, seafood will be an even more important source of food protein than it is today [42]. Thus, the safety of products from aquaculture for human consumption is a public health issue [56]. On the other hand, there are abundant metal residues in the fish flesh, as reflected by the high metal concentrations recorded in water and sediments [57]. Consequently, the health risks associated with the consumption of fish contaminated by heavy metals are becoming a significant worldwide concern [36].

The general population is primarily exposed to heavy metal ions via food [23]. For instance, fishes are the major source of methylmercury and dental amalgam [19]. However, the general population does not face a significant health risk from methylmercury, although certain groups, especially with regional high fish consumption, may attain blood levels associated with a low risk of neurodegenerative abnormalities or damage [5, 13, 29, 31]. Few metals, e.g. aluminum, can be removed through elimination activities, while some metals are accumulated in the body and food chain, exhibiting a chronic nature [21]. Metal toxicity depends upon the absorbed dose, the route of exposure, and the duration of exposure, i.e. acute or chronic [26, 27]. This can lead to various disorders and result in excessive damage due to oxidative stress induced by free radical formation [21].

Several methods have been proposed for the estimation of the potential risks of heavy metals in fishes to human health [59]. The risks may be divided into carcinogenic and non-carcinogenic. For carcinogenic contaminants, the observed or predicted exposure concentrations are compared with thresholds for adverse effects, as determined by dose-effect relationships [45]. The probability risk assessment technique has been adopted by several researchers [15, 45, 55, 59] to fully utilize available exposure and toxicity data. However, these methods have only been used to quantify the health risks of carcinogenic pollutants. Current non-cancer risk assessment methods do not provide quantitative estimates of the probability of experiencing non-cancer effects from contaminant exposure. These methods are typically based on the Target Hazard Quotient (THQ) [59].

In recent decades, much attention has been paid to the study of essential and toxic trace element content in foodstuffs, as a result of the growing concern about the health benefits and risks of food consumption [1]. Furthermore, fishes have been used for many years to indicate the pollution status of water and are thus regarded as excellent biomarkers of metals in aquatic ecosystems [32]. Moreover, it is important to note the level of heavy metals in consumed fish to recognize the safety of fish protein supplied to consumers and to understand its harmful effects on individuals, populations, or ecosystems [1].

Studies on the assessment of both dietary nutrients and heavy metal concentrations in commonly consumed fish species are still required. Such research is important in order to understand proper toxicity and eventual effects on humans [4]. Therefore, in recent years, numerous studies have focused on potential risks of heavy metals to human health through fish consumption [1, 4, 47, 48, 53]. The majority of studies published to date predominantly focus on toxic metals in fish meat [30, 33, 58], vegetables, fruits [3, 20, 34], and cereal crops [37, 38, 43, 44] as well as their levels and comparisons to various allowable limits.

The common carp (*Cyprinus carpio*) is one of the most important fish species cultured in Poland. In the minds of Poles, carp is inseparably connected with the tradition of the Christmas Eve table. Although it has been known and bred in Poland for centuries, the species has gained real popularity as a dish served during Christmas. During the holiday season, the interest in carp among Polish consumers is by far the greatest [2, 46]. Its sale in Poland is characterized by significant seasonality. It is estimated that about 90% of the annual domestic carp production is sold 2 weeks before Christmas. Poland has become the largest producer of carp in the European Union, as the domestic production accounts for about 30% of Union production. The Czech Republic and Hungary are also significant carp producers. Poland produces up to 20,000 tons of carp annually, which are almost entirely intended for the domestic market. Moreover, 90% of the annual production of carp is sold in the 2 weeks before Christmas [46]. Carp production and catch in 2019 accounted for nearly 36% of catching freshwater fish in Poland. The carp production in Poland in 2020 was estimated at approx. 21,000 tons.

There is limited information on heavy metal concentrations and nutritional elements in selected cultured fish, i.e. the common carp. Moreover, due to the increasing anthropogenic and industrial stresses, continuous monitoring of essential minerals and heavy metals in commonly consumed cultured fish is required [48].

Thus, the goals of our study were (i) to determine the concentration of essential minerals in the muscle tissue of carp obtained from a pond culture (fish used for consumption); (ii) to compare the content of these minerals with the physiological norms of nutrition for the Polish population, Recommended Dietary Allowances (RDA), and Estimated Average Requirements (EAR); (iii) to study the non-carcinogenic and carcinogenic risk level related to exposure to heavy metals and arsenic by assessment of the Estimated target hazard quotients (THQ), total target hazard quotients (ΣTHQ).

## Materials and methods

### Sample collection and preservation

Fifteen adult common carps, i.e. fish that is widely consumed by the Polish population especially at Christmas, were collected from the ponds of the farm of Production-Commercial Enterprise “AQUAMAR” Sp. z o.o. (Bożanka, West-Pomeranian Voivodship, northern Poland). A total of 15 individuals were collected, wrapped in polyethylene bags, and transported to the Analytical Chemistry Laboratory, Department of Ecology and Environmental Protection, Collegium Medicum in Bydgoszcz, Nicolaus Copernicus University in Toruń (Poland). Immediately after transportation to the laboratory, the samples were washed with fresh water to remove the fouling substances. For chemical analysis, the muscle tissue was sampled above the lateral line near the dorsal fin. Samples of fish tissue were weighed directly into acid-washed Teflon vessels.

Next, a fragment of muscle tissue was removed from each sample and chopped into pieces with the aid of a steam cleaned stainless steel knife. The muscle tissues were then washed with deionized water, air-dried to remove extra water, and subsequently homogenized in a food processor; 100 g of test portions were stored at −20 °C.

## Analytical methods

### Sample digestion

Microwave digestion with the use of concentrated nitric acid and hydrogen peroxide was used for the decomposition of the dried animal tissue, which was weighed into reaction vessels. Briefly, 8 ml of 69–70% Baker Instra Analyzed grade nitric acid was added together with 2 ml of 30% analytical grade hydrogen peroxide. Then, the samples were microwaved for 5 min at 190 °C (ramping time 25 min.), 5 min at 200 °C (ramping time 5 min.), and finally, 5 min at 210 °C (ramping time 5 min) to ensure total decomposition of organic matter. The digested solution was transferred into disposable calibrated tubes and filled up to 50 ml with 0.05 μS/cm deionized water.

### Instruments and reagents

The ICP-MS technique was used for the quantitative analysis of trace elements. The Agilent 7500ce ISP-MS apparatus is fitted with a micro-mist nebulizer, a Peltier cooled double pass spray chamber and a peristaltic pump. Argon 5.0 (99,999% purity) was used as a carrier gas. The apparatus is also fitted with a torch with a “shield torch” system reducing the so-called “secondary discharge”, off-axis ion lenses, a reaction/collision chamber with hydrogen 6.0, and helium 6.0 (purity 99,9999%) as reaction/collision gasses for the elimination of interferences. The vacuum system consists of a rotary pump and a turbo-molecular pump. A quadrupole with hyperbolic rods is the mass separator. The detector gives the possibility to work in two modes: digital and analog, which facilitates work through nine orders of magnitude.

The ICP-MS 7500ce apparatus from Agilent equipped with a micro-mist type nebulizer, cooled thermoelectrically using the Peltier effect, and a double-pass type fog chamber was used for the determination. The sample pumping speed was between 0.01 and 0.05 rpm. Argon 5.0 with a high purity of 99.999% was used as the carrier gas. The camera is equipped with a quartz burner with the “shield torch” option to prevent the formation of the so-called “secondary discharge”, i.e. electrical discharges arising between plasma generated from argon and camera cones. Standard nickel sampler and skimmer cones were used as well as CE type ion lenses misaligned to better eliminate polyatomic interference.

The apparatus is also equipped with an ORS (octopole reaction system) type reaction chamber to eliminate the interference of both polyatomic and doubly charged ions. In the reaction chamber, hydrogen 6.0 and helium 6.0 (purity 99.9999%) were used as reaction gases to eliminate interference. The apparatus vacuum system consists of an oil pre-pump and a turbo-molecular pump. The mass splitter is a quadrupole equipped with hyperbolic rods to create the correct electric field. The detector, i.e. an electron duplicator with the possibility of working in the plus and analog modes, allows achieving a 9-row dynamic range. To maintain apparatus stability and minimize matrix effects, all determinations were made in the presence of ^45^Sc, ^89^Y, and ^159^Tb as an internal standard.

### Quality control programs

All determinations were made in the presence of ^45^Sc, ^89^Y, and ^159^Tb as an internal standard to minimize the matrix effect and ensure long-term stability. The above procedure was also performed for the blank samples for the control of contamination. Simultaneously, for every series of samples, certified reference material (NCS ZC73016 chicken) from the China National Analysis Center for Iron and Steel was used to ensure quality control requirements. Recoveries ranging from 90 to 110% were achieved for this material and uncertainty of measurement was established at 10%.

## Statistical analysis and health risk assessment

Results are given as mean ± S.E.M. (*n* = 15) and expressed in milligram per kilogram wet weight (w.w.). The initial preparation of intermediate calculations was performed using Microsoft Excel 13.0. The obtained results were statistically analyzed using the STATISTICA 13.3 software package (StatSoft, Krakow, Poland). Both the maximum and minimum concentrations of chemical elements in the muscle tissue were determined to compare with the levels in edible fish filets as stated in the Codex Alimentarius (FAO/WHO 1995, last amended in 2015) [7], the Commission Regulation (EC) No 1881/2006 and Commission Regulation (EC) No 466/2001 (EC 2006, later amended in 2014 and 2015) [9, 10], and the Norms of nutrition for the Polish population [22].

The actual daily intake of nutrients through consumption of 100 g of a fish product by an adult weighing 70 kg in comparison with the allowable daily intake (ADI) was assessed according to FAO Fisheries Circular No 825 (Food Safety Regulation Applied to Fish Major Importing Countries, 1998) [14], Commission Regulation (EC) No 1881/2006 of 19 December 2006 setting maximum levels for certain contaminants in foodstuffs [9], Commission Regulation (EC) No 466/2001 of 8 March 2001 on the maximum levels for certain contaminants in foodstuffs [10], and the Regulation of the Minister of Health of Poland, 13.01.2003 on the maximum levels of chemical and biological contaminants that may be found in food, food ingredients, permitted additives, processing aids or on the surface of food (Journal of Laws 2003. No. 37, item 326 with later amendments) [39] to establish the same maximum levels of Hg, Cd, and Pb, i.e. 0.500, 0.050, and 0.300 mg kg^−1^ w.w., respectively.

For assessment of the potential risk of the dietary intake of mineral elements and heavy metals related to fish consumption, the recommended dietary allowances (RDAs), and Estimated Average Requirements (EAR) were used according to the Norms of nutrition for the Polish population [22]. The food and nutrition board of the Institute of Medicine suggests that the recommended dietary allowances (RDAs) or the adequate intakes (AIs) may be used as goals for individual intake as they are set to meet the needs of 97–98% individuals in a group.

The analysis of the data was based on FAO Fisheries Circular No 825 (Food Safety Regulation Applied to Fish Major Importing Countries, 1998) [14], Commission Regulation (EC) No 1881/2006 of 19 December 2006 on the maximum levels for certain contaminants in foodstuffs [9], Commission Regulation (EC) No 466/2001 of 8 March 2001 on the maximum levels for certain contaminants in foodstuffs [10], and the Regulation of the Minister of Health of Poland of 13.01.2003 on the maximum levels of chemical and biological contaminants that may be found in food, food ingredients, permitted additives, processing aids or on the surface of food (Journal of Laws 2003. No. 37, item 326 with later amendments) [39], USEPA (1986, 1989, 2000, 2010), i.e. Risk assessment guidance for Superfund, Human Health Evaluation Manual, EPA/540/1–89/002, Risk-Based Concentration Table, and Risk-based Concentration Table [40, 49–52].

### Estimated average daily dose (ADD) and daily intake (EDI)

The estimated average daily dose (ADD) and daily intake (EDI) of each heavy metal were calculated in the following way:

1 $$ \mathrm{ADD}=\left(\mathrm{EF}\times \mathrm{ED}\times \mathrm{C}\mathrm{R}\times \mathrm{C}\right)/\left(\mathrm{BW}\times \mathrm{TA}\right), $$

where ADD – an average daily dose of metal intake via the oral route (mg·kg^−1^);

EF – exposure frequency (365 days·year^−1^);

ED – exposure duration (70 years) equivalent to the average lifetime;

CR – the value of the contact, i.e. the amount of contaminated substance in contact with the human body at the daily intake (for products – kg·person^−1^·day^−1^);

C – metal concentration in samples (mg·kg^−1^);

BW – average body weight (kg);

TA – averaging exposure time for noncarcinogens (365 days·year^−1^·number of exposure years) [54].

The estimated daily intake (EDI) of each heavy metal was calculated according to Saha and Zaman (2013) [41]:

2 $$ \mathrm{EDI}=\frac{E_{\mathrm{F}}\times {E}_{\mathrm{D}}\times {F}_{\mathrm{IR}}\times {C}_{\mathrm{f}}\times {C}_{\mathrm{m}}}{W_{\mathrm{A}\mathrm{B}}\times {T}_{\mathrm{A}}}\times {10}^{-3}, $$

where E_F_ – exposure frequency (365 days·year^−1^);

E_D_ – exposure duration equivalent to average lifetime;

F_IR_ – fresh food ingestion rate (g·person^−1^·day^−1^);

C_f_ – conversion factor (0.208) used to convert fresh weight (f.w.) to dry weight (d.w.) considering 79% of moisture content;

C_m_ – heavy metal concentration in foodstuffs (mg·kg^−1^d.w.);

W_AB_ – average body weight (B.W.) (the average adult body weight was considered to be 60 kg);

and T_A_ – average exposure time for non-carcinogens (equal to E_F_ × E_D_) as used in many previous studies [54].

### Non-carcinogenic risk

The non-carcinogenic health risks associated with the consumption of fish were assessed based on the target hazard quotients (THQs). The THQ values related to the consumption of fish species can be assessed for each heavy metal, and calculations were made using the standard assumption for an integrate USEPA risk analysis as follows [1, 50]:

3 $$ THQ=\frac{EFr\times ED\times FIR\times C}{RfD\times BW\times AT}{10}^{-3} $$

where THQ – target hazard quotient (dimensionless);

EFr – exposure frequency (365 days·year^−1^);

ED – exposure duration (70 years) equivalent to the average human lifetime;

FIR – food ingestion rate (g·person^−1^·days^−1^);

C – metal concentration in samples (mg kg^−1^, wet weight);

BW – average body weight (adult, 60 kg);

AT – averaging time for non-carcinogens (365 days·year^−1^·number of exposure years, assuming 70 years).

RfD – oral reference dose (mg kg^−1^ d^−1^);

RfDs are based on 0.001, 0.0003, 0.004, 1.5, 0.02, and 0.04 mg·kg^−1^ BW d^−1^ for Cd, As, Pb, Cr, Ni, and Cu, respectively [52]. RfDs represent an estimate of the daily exposure to which the human population may be continually exposed over a lifetime without an appreciable risk of deleterious effects. If the THQ is less than 1, the exposed population is unlikely to experience obvious adverse effects. If the THQ is equal to or higher than 1, there is a potential health risk [54] and related interventions and protective measurements should be taken.

### Combined risk of multiple heavy metals

It has been reported that exposure to two or more pollutants may result in additive and/or interactive effects [17, 41]. The total THQ (TTHQ) of heavy metals for individual foodstuff was treated as the mathematical sum of the THQ value of each metal:

4 $$ \mathrm{TTHQ}\ \left(\mathrm{individual}\ \mathrm{foodstuff}\right)=\mathrm{THQ}\ \left(\mathrm{toxicant}1\right)+\mathrm{THQ}\ \left(\mathrm{toxicant}\ 2\right)+\dots +\mathrm{THQ}\ \left(\mathrm{toxicant}\ \mathrm{n}\right). $$

To assess the overall potential risk of non-carcinogenic effects posed by more than one element, the Hazard Index (HI) approach has been developed by USEPA (1986) [49]. The HI for a specific receptor/pathway combination (e.g., diet) was calculated as follows:

5 $$ \mathrm{HI}=\mathrm{TTHQ}\ \left(\mathrm{foodstuff}\ 1\right)+\mathrm{TTHQ}\ \left(\mathrm{foodstuff}\ 2\right)+\dots +\mathrm{TTHQ}\ \left(\mathrm{foodstuff}\ \mathrm{n}\right). $$

When the HI exceeds unity, there may be a concern for potential health risks.

### Carcinogenic risk

For carcinogens, the risks were estimated as the incremental probability of an individual developing cancer over lifetime exposure to a potential carcinogen (i.e., incremental or excess individual lifetime cancer risk [50]. Acceptable risk levels for carcinogens range from 10^-4^ (the risk of developing cancer over a human lifetime is 1 in 10,000) to 10^–6^ (the risk of developing cancer over a human lifetime is 1 in 1,000,000). The equation used for estimating the target cancer risk (lifetime cancer risk) is as follows [50]:

6 $$ TR=\frac{EFr\times ED\times FIR\times C\times CSFo}{BW\times AT} $$

where TR represents the target cancer risk or the risk of cancer over a lifetime; CSFo is the oral carcinogenic slope factor from the Integrated Risk Information System [52] database (1.5 mg·kg^−1^·days^−1^ for arsenic and 0.0085 mg·kg^−1^·days^−1^ for lead).

Carcinogenic risk (CR) indicates an increased likelihood of an individual developing life-threatening cancer that is due to exposure to a potential carcinogen. The level used to assess the risk of cancer [51] is as follows:

7 $$ \mathrm{CR}=\mathrm{ADD}\cdotp \mathrm{CSF}, $$

where CR – carcinogenic risk; CSF – carcinogenic factor tilt.

CSF is the carcinogenic slope factor of 0.0085 mg·kg^−1^·day^−1^ for Pb and 1.5 mg·kg^−1^·day^−1^ by USEPA (2010) [52]. EDI is the estimated daily intake of heavy metals. Acceptable risk levels for carcinogens range from 10^−4^ (the risk of developing cancer over a human lifetime is 1 in 10,000) to 10^−6^ (the risk of developing cancer over a human lifetime is 1 in 1,000,000). The risk of cancer benchmark (1·10^−6^) is often used by EPA as the lower end of the range of acceptable risk [18].

## Results and discussion

The mean contents of minerals (manganese, iron, copper, zinc, magnesium, calcium, cobalt, sodium, selenium, phosphorus, potassium, aluminum, chromium) with a range of minimum and maximum values in the muscle tissue of carp are summarized in Table 1. The chemical analyses indicated that the muscle tissue of the common carp was rich in sodium (206.54 ± 31.938) mg·kg^−1^, potassium (97.88 ± 11.243) mg·kg^−1^, calcium (78.40 ± 17.721) mg·kg^−1^, phosphorus (48.47 ± 7.387) mg·kg^−1^, and magnesium (27.07 ± 4.523) mg·kg^−1^ (Table 1). The concentrations of other micro- and macro-elements in the samples were negligible, as they were less than 1 mg·kg^−1^. The pattern significance gradation of the element concentration was as follows: Na > K > Ca > P > Mg > Al > Zn > Fe > Cu > Mn > Cr > Se > Co.

**Table 1:** Concentrations of elements in the muscle tissue of the common carp (*n* = 15)

Elements	Concentration (М ± m), mg·kg^−1^	Range of values (min-max), mg·kg^−1^
Manganese, Mn	0.017 ± 0.003	0.006–0.028
Iron, Fe	0.368 ± 0.048	0.187–0.546
Copper, Cu	0.035 ± 0.007	0.012–0.064
Zinc, Zn	0.726 ± 0.121	0.358–1.17
Magnesium, Mg	27.07 ± 4.523	5.9–43.1
Calcium, Ca	78.40 ± 17.721	11.6–146.0
Cobalt, Co	0.0003 ± 0.00005	0.0001–0.0005
Sodium, Na	206.54 ± 31.938	15.2–266.0
Selene, Se	0.005 ± 0.0004	0.003–0.007
Phosphorus, P	48.47 ± 7.387	17.6–78.9
Potassium, K	97.88 ± 11.243	54.7–139.0
Aluminum, Al	0.943 ± 0.108	0.43–1.5
Chrome, Cr	0.012 ± 0.0004	0.01–0.013

The human requirement for nutrients derived from foods was assessed according to the nutritional standards for the Polish population [22], comparing the actual intake of nutrients with the Recommended Dietary Allowances (RDA) and Estimated Average Requirements (EAR) (Table 2). According to the Food and Nutrition Board of the Institute of Medicine, the Recommended Dietary Allowances (RDA) reflect an average daily intake that is sufficient to meet the nutrient requirements of nearly all (97%–98%), healthy people in a particular gender and life stage group [12]. The RDA for a nutrient is a value to be used as a goal for dietary intake by healthy individuals. The Estimated Average Requirements (EAR) is the daily intake value of a nutrient that is estimated to meet the nutrient requirement of half the healthy individuals in a life stage and gender group [22].

**Table 2:** Potential risk of mineral intake through consumption of 100 g of common carp compared to the Recommended Dietary Allowances (RDA) (*n* = 15)

Elements	Recommended Dietary Allowances (RDA^a^), mg·day^−1^·person^−1^		Actual intake of minerals from 100 g of product, mg·day^−1^·person^−1^	Potential risk of mineral intakes according to RDA^a^, (%)	
	males	females		males	females
Manganese, Mn	2.3	1.8	0.002	0.09	0.11
Iron, Fe	10.0	18.0	0.037	0.37	0.21
Copper, Cu	0.9	0.9	0.003	0.33	0.33
Zinc, Zn	11.0	8.0	0.073	0.66	0.91
Magnesium, Mg	420.0	320.0	2.707	0.64	0.84
Calcium, Ca	1000.0	1200.0	7.84	0.78	0.65
Selene, Se	5.5	5.5	0.0005	0.009	0.009
Phosphorus, P	700.0	700.0	4.847	0.69	0.69

^a^RDA according to Jarosz M. (eds.) Norms of nutrition for the Polish population. IŻŻ, Warsaw, 2017 (in Polish)

In the current study, the actual intakes of micro- and macro-elements through the daily consumption of 100 g of carp flesh meeting the nutritional requirements of adults (a mean bodyweight of 70 kg) aged 18 years and older were analyzed. The potential risk of intake of chemical elements through carp consumption was calculated based on the average actual daily intake concerning existing standard levels (RDA and EAR) according to Commission Directive 2008/100/EC of 28 October 2008 amending Council Directive 90/496/EEC on nutrition labeling for foodstuffs as regards recommended daily allowances, energy conversion factors, and definitions [8], as well as the Norms of nutrition for the Polish population [22]. The potential risk of the actual intake of micro- and macro-elements to adults (both males and females) with 100 g of carp flesh compared to the RDA and AEQ is presented in Tables 2 and 3. This comparison shows that the potential risk is relatively low.

**Table 3:** Potential risk of mineral intake with 100 g of carp flesh compared to the Estimated Average Requirements (EAR) (*n* = 15)

Elements	Estimated Average Requirements (EAR)^a^, mg·kg^−1^		The potential risk of mineral intakes according to EAR^a^, (%)	
	males	females	males	females
Manganese, Mn	2.3	1.8	0.09	0.11
Iron, Fe	6.0	6.0	6.17	6.17
Copper, Cu	0.7	0.7	0.43	0.43
Zinc, Zn	9.4	6.8	0.78	1.07
Magnesium, Mg	350.0	265.0	0.77	1.02
Calcium, Ca	800.0	800.0	0.98	0.98
Selene, Se	4.5	4.5	0.01	0.01
Phosphorus, P	580.0	580.0	0.83	0.83

^a^EAR according to Jarosz M. (eds.) Norms of nutrition for the Polish population. IŻŻ, Warszawa, 2017 (in Polish)

Our study revealed (Table 2) that the consumption of 100 g of carp flesh can provide an adult with low quantities of minerals, i.e. manganese, iron, copper, zinc, magnesium, calcium, selenium, and phosphorus (0.09% and 0.11%, 0.37% and 0.21%, 0.33% and 0.33%, 0.66% and 0.91%, 0.64% and 0.84%, 0.78% and 0.65%, 0.009% and 0.009%, 0.69% and 0.69% of the RDA for males and females, respectively) according to the Norms of nutrition for the Polish population [22] and the EU Commission Directive (2008) [8]. These elements were present in a low concentration in the carp flesh and probably did not significantly affect its nutritional value.

The potential risk of the intake of minerals from 100 g of carp in comparison with the Estimated Average Requirements (EAR) is presented in Table 3.

The highest intake of zinc and magnesium (1.07% and 1.02%, respectively) compared to the EAR was observed in the group of females after consumption of 100 g of carp flesh (Table 3). The consumption of 100 g of carp flesh can provide the other micro- and macro-elements studied in quantities lower than 1% of the EAR for the male and female population according to the Norms of nutrition for the Polish population [22]. The consumption of carp as a food product with the contents of these minerals can only be a supplement to other diets because these trace elements were found in small quantities in the samples studied.

The hygienic regulation of the xenobiotic contents in foods requires compliance with two types of standards: Maximum Permissible Concentration (MPC) or Maximum Permissible Level (MPL) in individual products as well as Acceptable Daily Intake (ADI). The standards are the basis for performing hygienic control of chemical levels in food raw materials and finished products. The norm of the MPC and MPL are the criteria for the safety of individual food products, while ADI reflects the dietary standards for the population.

The concentrations of heavy metals, Pb, Cd, As, and Hg in carp flesh are listed in Table 4. All metal concentrations were determined on a wet weight basis. The heavy metal concentrations in the fish samples were estimated at 0.00004 ± 0.00025 mg kg^−1^ for Cd, 0.00247 ± 0.10462 mg kg^−1^ for Pb, 0.0003 ± 0.0006 mg kg^−1^ for Hg, and 0.0009 ± 0.0739 mg kg^−1^ for As. According to these results, the ranking order of the mean concentration of the heavy metals in the carp muscles was Pb (0.0362 mg·kg^−1^) ˃ As (0.0021 mg·kg^−1^) ˃ Hg (0.00051 mg·kg^−1^) > Cd (0.00014 mg·kg^−1^).

**Table 4:** Comparison of the concentration of heavy metals in the common carp with the maximum permissible level of metals in the product (*n* = 15)

Toxic metals	Concentration (М ± m), mg·kg^−1^	Range of values (min-max), mg·kg^−1^	Maximum permissible concentration (MPC^a^) of metals in the product, mg·kg^−1^
Cadmium, Cd	0.00014 ± 0.00003	0.00004–0.00025	0.05
Lead, Pb	0.0362 ± 0.0149	0.00247–0.10462	0.2
Mercury, Hg	0.00051 ± 0.00003	0.0003–0.0006	0.5
Arsenic, As	0.0021 ± 0.00015	0.0009–0.0739	4.0

^a^ The Maximum Permissible Concentration (MPC) is the maximum quantity of an injurious substance per unit volume (air, water, or other liquid) or weight (for example, food products) to which daily exposure for an indefinite period does not cause any pathological deviations or unfavorable hereditary changes in offspring

Toxic metals, i.e. cadmium, lead, mercury, and arsenic, were found in the studied fish samples (Table 4). The contents of the heavy metals in the carp flesh were compared to the maximum level of metals in animal-origin products vs. the restrictions of the European Union submitted by the Commission Regulation (EC) No 1881/2006 [9] and Commission Regulation (EC) No 466/2001 [10]. The concentration of heavy metals in the samples was lower than the maximum permissible level for metals in food products (Table 4).

The estimated daily intake of toxic metals with carp muscle tissue (cadmium, lead, mercury, and arsenic) in the human body is presented in Table 5.

**Table 5:** Estimated daily intake and risk of daily intake of toxic metals to the human body through consumption of carp flesh

Toxic metals	Estimated daily intake of heavy metals from 100 g of product, mg·day^−1^·person^−1^	Acceptable Daily Intake (ADI^a^), mg·day^−1^·person^−1^	Recommended daily dietary allowance (RDA^b^), mg·day^−1^·person^−1^	Risk of the toxic metal intake according to ADI, %	Potential risk of the toxic metal intakes according to RDA, %
Cadmium, Cd	0.000014	0.07	0.06	0.02	0.023
Lead, Pb	0.00362	0.25	0.21	1.45	1.73
Mercury, Hg	0.000051	0.05	0.03	0.1	0.17
Arsenic, As	0.00021	1.05	0.13	0.02	0.16

^a^ The Acceptable Daily Intake (ADI) is defined as the maximum amount of a chemical that can be ingested daily over a lifetime with no appreciable health risk and is based on the highest intake that does not give rise to observable adverse effects

^b^ The recommended daily dietary allowance (RDA) is the average daily level of intake sufficient to meet the nutrient requirements of nearly all (97%–98%) healthy people. According to the JECFA, Evaluations of the Joint FAO/WHO Expert Committee on Food Additives, 2009 [24]

The results shown in Table 5 revealed the lowest daily intake of Cd (0.000014 mg·day^−1^·person^−1^) and the highest daily intake of Pb (0.00362 mg·day^−1^·person^−1^). The estimated daily intake was calculated by considering that a 60-kg person consumes 100 g fish per day. It was revealed that the EDI values for the examined fish samples were below the recommended values, which indicated no risk to health associated with the intake of the studied heavy metals through the consumption of the carp flesh.

The actual daily intake of chemicals through consumption of 100 g of this food product by an adult in comparison with the allowable daily intake (ADI) was assessed according to FAO Fisheries Circular No 825 (Food Safety Regulation Applied to Fish Major Importing Countries, 1998) [14], Commission Regulation (EC) No 1881/2006 of 19 December 2006 setting maximum levels for certain contaminants in foodstuffs [9], Commission Regulation (EC) No 466/2001 of 8 March 2001 setting maximum levels for certain contaminants in foodstuffs [10], and Regulation of the Minister of Health of Poland, 13.01.2003 on the maximum levels of chemical and biological contaminants that may be found in food, food ingredients, permitted additives, processing aids or on the surface of the food [39]. Based on the mean content of toxic substances, the actual daily intake, and the acceptable daily intake, the potential risk of heavy metal intakes through consumption of 100 g of carp flesh was calculated and presented in Table 5. Thus, the risk of toxic metal intakes for an adult with daily consumption of 100 g of carp flesh was 1.45% for Pb, 0.1% for Hg, 0.02% for Cd, and 0.02% for As, compared to the acceptable daily intake for each toxicant (Table 5).

The assessment of the non-carcinogenic risk is usually carried out to determine the health effects of pollutants constituting a potential hazard (Ullah et al., 2017). Health risks were also assessed by determining the Target Hazard Quotient (THQ) of each heavy metal as well as the Hazard Index (HI). The THQ estimates the noncarcinogenic risk level related to the consumption of a specific pollutant present in a product, while the HI, i.e. the sum of THQs, estimates the global risk related to the consumption of the product [28].

The results shown in Table 6 revealed that the THQ value of each metal was less than 1, suggesting that individuals would not experience significant health risks at an intake of each heavy metal alone through the consumption of farmed carp flesh. Also, the total target hazard quotient (ΣTHQ) was less than 1, indicating that there was no considerable health hazard through the consumption of carp flesh and exposure to a mixture of the four studied metals (As, Pb, Cd, Hg). In the current study, the major risk contributor was Pb with 82.9%, followed by Hg (9.38%), As (6.43%), and Cd (1.29%).

**Table 6:** Estimated target hazard quotients (THQ), total target hazard quotients (ΣTHQ), and carcinogenic risk of each metal caused by the consumption of the carp flesh

Toxic metals	Average daily dose (ADD), mg·kg^−1^·d^−1^	Oral reference dose (RfD), (mg·kg^−1^·d^−1^)	Target hazard quotients (THQ)	Total target hazard quotients (ΣTHQ)	Carcinogenic risk (CR)
Cadmium, Cd	2.0^*^10^−8^	0.001	2.0^*^10^−5^	1.56^*^10^−3^	
Lead, Pb	5.17^*^10^−6^	0.004	1.29^*^10^−3^		4.39^*^10^−8^
Mercury, Hg	7.28^*^10^−8^	0.0005	1.46^*^10^−4^		
Arsenic, As	3.0^*^10^−7^	0.003	1.0^*^10^−4^		4.5^*^10^−7^

The total THQ value was also lower than 1, indicating the safety of the combined effects of chemicals. The carcinogenic risk (CR) was 4.39·10^−8^ for Pb and 4.5·10^−7^ for As, indicating a negligible level of cancerogenic risk. Usually, the values of CR lower than 10^−6^ are regarded as negligible, those above 10^−4^ are unacceptable, and values between 10^−6^ and 10^−4^ are regarded as an acceptable range [52]. In the present study, the CR for Pb and As was lower than the unacceptable range, indicating that the risk of cancer related to exposure to those heavy metals through fish consumption was negligible.

It may be mentioned that only fish consumption was considered in the determination of the health risk associated with the intake of trace metals. We can thus suggest that other food sources, particularly vegetables, fruits, cereals, and piscine and non-piscine protein sources need to be considered to evaluate the exact health risks related to the dietary intake of trace metals. Moreover, constant monitoring of heavy metals in all food commodities is necessary to evaluate if there are any potential health risks of heavy metal exposure, to assure food safety, and to protect end users from food that might deteriorate their health.

Consequently, the consumption of the studied carp flesh from fish farms by individuals does not create health risks or pose a considerable health hazard. Nevertheless, the potential health risk for individuals through the consumption of fish with high metal levels should not be ignored. Similarly, other sources of metal exposure, such as consumption of other foodstuffs and dust inhalation, which are not included in this study, should not be neglected. Therefore, we suggest that constant monitoring of both toxic and nutrient elements in all food commodities is indispensable for evaluation of the existence of any potential health risks.

Similar investigations were conducted on cultured and wild fish and the corresponding health risks through fish consumption. For example, Jiang and co-workers (2014) have evaluated the levels of heavy metals in fish (6 big-head carps, 5 grass carps, 5 carps, and 5 tilapias) from aquaculture farms and assessed the risk in Lhasa, Tibetan Autonomous Region of China. The contents of toxic metals (As, Cd, and Pb) in the liver were higher than in other tissues, whereas the heavy metal levels in the muscle were the lowest among the different tissues. Besides, Cr, Ba, Co, Mn, and V tended to accumulate in the gill, and Cu was highly accumulated in the hearts of fish. While estimating the daily intake of As, Cd, and Pb through fish consumption by the local inhabitants, it was concluded that the daily intake of these metals in this area did not exceed the TDI recommended by FAO [25].

The distribution of toxic elements in cultured and wild fish and the corresponding health risks through fish consumption in the Honghu area were investigated by Zhang and co-workers (2018) [60]. The mean concentration in the muscle of cultured and wild fish (*Carassius auratus* and *Ctenopharyngodon idellus*) decreased in the following order: Zn (18.94) > Cu (0.8489) > Cr (0.2840) > Pb (0.2052) and Zn (16.30) > Cr (1.947) > Cu (0.4166) > Pb (0.0525) > Cd (0.0060) (mean; mg/kg, wet weight). No obvious health risks associated with the consumption of cultured and wild fish were demonstrated by the calculated results of the THQ, carcinogenic risk (CR), an estimated weekly intake (EWI). Pb and Cr were recognized as the major health risk contributors for inhabitants through the consumption of the wild and cultured fish. The cultured fish posed a greater health risk than the wild fish based on the results of THQ and CR. The consumption of the muscles resulted in higher health risks than the consumption of mixed edible tissues of the cultured fish; the opposite was found in the case of the wild fish [60].

On the other hand, Varol and co-workers (2019) determined the concentrations of 10 heavy metals and one metalloid (As) in samples of ten different fish species sold in Turkey to assess human health risks associated with fish consumption and to compare the results with those from other studies and with the maximum permissible limits set by various international standards. The lowest and highest toxic metal contents were recorded in the rainbow trout and the red mullet, respectively, while the lowest and highest essential metal contents were found in the red mullet and the European anchovy. The mean As content in the red mullet was 209.8-fold higher than that of the rainbow trout. The human health risk assessment indicated that individual and combined metals in fish species did not pose non-carcinogenic risks to people. However, inorganic As in the red mullet would cause potential carcinogenic risk to consumers. Among fish species, freshwater fish species were safer than other species in terms of human health [53].

The potential health risk of heavy metals to local residents at long-term carp consumption has been demonstrated in some studies. Investigations of the heavy metal contents in different tissues (gill, liver, intestine, and muscle) of the common carp and use of these data to estimate the health risk of heavy metal pollution in the upper Mekong River section under the influence of the cascade dams (western Yunnan province, China) was performed by Zhang and co-workers (2019). The highest Cu and As levels were found in the liver; the highest Zn and Pb levels were detected in the intestine, and the highest Hg level was found in the muscle. The total target hazard quotient (THQ) value for residents is >1 for long-term fish consumption; therefore, local residents are exposed to a significant health risk [61]. Gwimbi and co-workers (2020) assessed the concentration of heavy metals (As, Pb and Zn) in sediments and gills of common carp fish from Maqalika Reservoir in Maseru, Lesotho, and their potential health risks to consumers of such fish. The As and Pb levels in the gills of carp exceeded the permissible limit recommended for fish consumption by FAO/WHO [16]. The concentrations of arsenic, cadmium, mercury, and lead in 149 muscle samples of eight freshwater fish species (European eel, bream, common carp, European catfish, roach, perch, pike, and pikeperch) from five different French fishing areas from contaminated and control sites were studied by Noël and co-workers (2013). Significant differences in the Hg and Pb levels between groups of predatory fish and non-predatory fish and between control and contaminated sites in the whole selection as well as within feeding guilds were reported by these researchers [35].

## Conclusions

1. The results of chemical analyses indicated that the muscle tissue of the farmed common carp was rich in sodium, potassium, calcium, phosphorus, and magnesium. The concentrations of other micro- and macro-elements in the samples were insignificant, i.e. less than 1 mg·kg^−1^. The pattern significance gradation of element concentrations was as follows: Na > K > Ca > P > Mg > Al > Zn > Fe > Cu > Mn > Cr > Se > Co.

2. The consumption of 100 g of farmed common carp can provide an adult with minor quantities of nutrient minerals (Mn, Fe, Cu, Zn, Mg, Ca, Se, P), i.e., 0.009–0.84% of the Recommended Dietary Allowances for minerals for males and females, respectively according to the Norms of nutrition for the Polish population (2017) and the EU Commission Directive (2008). These microelements were present in a low concentration in the carp muscle tissue and did not significantly affect its nutritional value.

3. The estimation of toxic metals in the farmed carp revealed that the mean content of these metals did not exceed the maximum permissible level. The concentration of toxic heavy metals in the samples was lower than the maximum allowable level of the metals in food products. The risk of consumption of toxicants by an adult at a daily intake of 100 g of fish was 1.45% for Pb, 0.1% for Hg, 0.02% for Cd, and 0.02% for As, compared to the allowable daily intake for each toxicant.

4. The value of the target hazard quotients of each metal was less than 1, indicating that the intake of a single metal through the consumption of carp meat does not pose a considerable health hazard. The total value of the target hazard quotients was also less than 1, indicating the safety of the combined effects of chemicals. The potential health risk related to the exposure to a mixture of the four studied metals (As, Pb, Cd, Hg) through consumption of carp muscle tissue was negligible. In the current study, the major risk contributor was Pb with 82.9%, followed by Hg (9.38%), As (6.43%), and Cd (1.29%).
